# The Polish vocabulary size test: A novel adaptive test for receptive vocabulary assessment

**DOI:** 10.3758/s13428-025-02775-3

**Published:** 2025-08-11

**Authors:** Danil Fokin, Monika Płużyczka, Grigory Golovin

**Affiliations:** 1 University of Warsaw, Warsaw, Poland; 2 Independent, San Jose, California USA

**Keywords:** Polish vocabulary, Vocabulary test, Language proficiency testing, Computerized adaptive testing, Item response theory, Rasch modelling

## Abstract

We present the Polish Vocabulary Size Test (PVST), a novel tool for assessing the receptive vocabulary size of both native and non-native Polish speakers. Based on item response theory and computerized adaptive testing, PVST dynamically adjusts to each test-taker’s proficiency level, ensuring high accuracy while keeping the test duration short. To validate the test, a pilot study was conducted with 1475 participants. Native Polish speakers demonstrated significantly larger vocabularies (mean = 75,125 words; range = 19,556–122,693) compared to non-native speakers (mean = 7165 words; range = 646–23,394). For native speakers, vocabulary size showed a strong positive correlation with age (r = .496, p < .001). The PVST is available online at myvocab.info/pl.

## Introduction

Vocabulary size is a crucial component of language proficiency (Laufer & Nation, 2001; Qian & Schedl, 2004), enhancing reading comprehension, proficiency, and efficiency (Engku et al., 2016; Chateau & Jared, 2000; Masrai, 2019; Schmitt, 2014; Tschirner, 2021), as well as speed of word and meaning recognition (Allal-Sumoto et al., 2024; Laufer & Nation, 2001; Lemhöfer et al., 2008). However, vocabulary is not a homogeneous continuum, being categorized into receptive and productive types (Laufer & Goldstein, 2004; Laufer & Paribakht, 1998; Webb, 2005). Since receptive vocabulary is acquired by reading and listening and refers to form-to-meaning mapping (Laufer & Paribakht, 1998; Schmitt, 2014), most vocabulary size tests are aimed at assessing the passive part of the lexicon (Stoeckel et al., 2021; Webb, 2021).

Both receptive and productive vocabularies are modulated by many factors, e.g., education, time spent reading/listening, or multilingualism (Guasch et al., 2023; Mainz et al., 2017; Kuperman & Van Dyke, 2013; Vermeiren et al., 2023). Age of acquisition has shown a consistent correlation with vocabulary size among native speakers in Dutch (Keuleers et al., 2015), English (Brysbaert et al., 2016), German (Boone & De Wilde, 2023), and Catalan (Guasch et al., 2023).

The effective vocabulary test is expected to distinguish natives and non-natives based on vocabulary breadth, and establish a correlation between vocabulary size and age for native speakers. In this paper, we present the pilot results of the Adaptive Polish Vocabulary Size Test (APVST), which aims to become a reliable tool for testing the receptive vocabulary of native and non-native Polish speakers.

## Literature review

### Vocabulary size tests

There are many ways to measure vocabulary size: paper-and-pencil tasks (e.g., Kavé & Halamish, 2015), subscales of Wechsler’s IQ test (Sohacka, 2016) and Nelson–Denny Reading test (Andrews et al., 2020; Vermeiren et al., 2023), Peabody Picture Vocabulary Test (Bohn et al., 2024; Carroll et al., 2016; Mainz et al., 2017), or Vocabulary Levels Test (Schmitt et al., 2001). Nowadays, the two most popular ones are the Vocabulary Size Test (VST) (Nation & Beglar, 2007) and LexTale (Lemhöfer & Broersma, 2012).

VST has versions to examine receptive or productive vocabulary (Nation, 2024), and adapted to Japanese (Derrah & Rowe, 2015), Korean (Park, 2024), Mandarin (Zhao & Ji, 2018), and Persian (Karami, 2012). In the VST, items are clustered based on frequencies, and participants select either a synonym or word definition out of the list of items. LexTale was translated into German, Dutch, and English (Lemhöfer & Broersma, 2012), French (Brysbaert, 2013), Italian (Amenta et al., 2021), Spanish (Ferré & Brysbaert, 2017), Portuguese (Zhou & Li, 2022), and Chinese (Chan & Chang, 2018; Qi et al., 2022). It is based on the lexical decision task, where participants decide whether the string of letters is a real word.

Both tests are noted to have limitations. Coxhead et al. (2014) argued that guessing in the multiple-choice task (main VST principle) may inflate scores in the meaning recognition task and overestimate vocabulary knowledge. Stoeckel et al. (2021) mentioned the scoring interpretation, number and types of items are critical shortcomings of the tests that lead to vocabulary size overestimation (for further discussion, see Webb, 2021; Read, 2023). LexTale was criticized for being overestimated in its reliability, while the original study by Lemhöfer & Broersma (2012) lacks replications and is not well suited to capture differences in second language proficiency (Puig-Mayenco et al., 2023).

The alternative to the above-mentioned methods is computerized adaptive testing (CAT), which is based on item response theory (IRT). The popularity of this method is rising (Bohn et al., 2024; Chan & Chang, 2018; Gibson & Stewart, 2014; Tseng, 2016). The main advantage of this approach is the automatic selection of the upcoming stimulus based on the participant’s previous response, which increases reliability and precision while minimizing time- and cognitive-consumption. Based on this methodology, Golovin (2015) developed an Adaptive online Vocabulary Size Test (AoVST) for the Russian language. AoVST demonstrated strong nonlinear relations between receptive vocabulary and age and high validity based on more than 400,000 responses. AoVST is adapted to English, German, Ukrainian, Tatar, and Hebrew and has been used in several psycholinguistic studies (Maslennikova et al., 2017; Parshina et al., 2024; Westergaard, 2024). A similar approach is employed in the present study.

### Vocabulary testing in Polish

For Polish, the number of tools to examine one’s vocabulary size is limited. One of the methods used is a verbal subscale of Wechsler’s test for adolescents (WAIS-R). However, it is only a part of a cognitive battery, being not designed to measure vocabulary exclusively. Sochacka (2019) indicated WAIS-R volatility due to the Flynn effect, i.e., “the gradual cross-cultural rise in raw scores obtained on measures of general intelligence” (American Psychological Association, 2018a) and mentioned that test-takers of the children’s version (WISC-R) outperform adolescents in some subscales (Sohacka, 2019). Thus, the Polish adaptation of Wechsler’s test is outdated, and its reliability is understudied.

Recently, Muszyński et al. (2023) introduced an adaptation of the Pathfinder General Cognitive Ability Test (PGCAT), which includes verbal and vocabulary subscales, as well as an unpublished version of LexTale-PL. PGCAT demonstrated high validity and precision in intelligence testing. However, the vocabulary subscale is unlikely to be applicable for size testing. Researchers do not separate productive and receptive vocabularies and employ a non-standardized approach for stimulus and synonym selection, including direct translations from English, equivalent substitutions, brainstorming sessions, and reliance on frequencies extracted from Duolingo. This lack of transparency may potentially undermine the validity of the stimulus selection method, rendering the vocabulary subscale insensitive to vocabulary size differences.

### The current study

The goal of the present study is to bridge the gap in Polish vocabulary testing by developing a tool that is both reliable and requires little time and cognitive effort. We developed an adaptive Polish Vocabulary Size Test (PVST), which is focused on testing receptive vocabulary and language proficiency rather than cognitive constructs, making it relevant for linguistic research. Using adaptive testing, we addressed the drawbacks of other tests. To validate the test, we formulated the following research questions:

1. Is the item pool used in PVST sufficiently representative for evaluating the receptive vocabulary size of Polish language users?
2. Does the methodology used effectively capture underlying cognitive processes associated with vocabulary knowledge?
3. Is PVST reliable across different age groups, native and non-native speakers?

### Test design

The Polish Vocabulary Size Test (PVST) is based on item response theory (IRT) (Reise & Waller, 2009; Embretson & Reise, 2000) and computerized adaptive testing (CAT) (Straetmans & Eggen, 1998; Eggen, 2018). It uses binary (yes/no), multiple-choice, and pseudoword stimuli (see *Stimuli types* and *Stimuli selection* sections for more details). A stimulus is presented and assesses whether the test-taker knows that word. Based on the responses, we estimate the test-taker’s ability and its associated uncertainty using standard IRT procedures (see the *Scoring* section for details). We then followed a CAT approach, selecting the next test item to match its difficulty to the test-taker’s estimated ability level. The process continues until 30 stimuli have been presented. We conducted four pilot tests until we found the number of stimuli that strikes a good balance between the duration of the test (approximately 2 minutes) and its precision and accuracy (see the Results – *Rasch analysis section* for details). Finally, we convert the estimated ability from logits (IRT units) to words, making the results more meaningful for test-takers. We also compute an attention index, which serves as a measure of result trustworthiness (see the *Scoring* section for further details).

Computerized adaptive testing, which is central to the PVST, has proven to be a powerful approach to vocabulary assessment, offering efficiency, precision, and a customized experience unmatched by traditional tests. Across diverse populations, CAT-based vocabulary tests deliver shorter yet equally reliable measures of vocabulary size (Mizumoto et al., 2017; Tseng, 2016). They achieve this through intelligent item selection tailored to each individual’s proficiency level, resulting in precise scores while minimizing unnecessary testing. Indeed, presenting simple stimuli to proficient native speakers or difficult stimuli to beginner learners would provide little useful information. By matching each stimulus to the current estimate of the test-taker’s ability, we maximize the amount of information each stimulus contributes to the test. Furthermore, the individualized nature of CAT broadens accessibility (one test fits all proficiency levels) and enhances the test-taking experience, potentially reducing anxiety related to overly easy or difficult stimuli and boosting participant engagement (Kachergis et al., 2022; Ling et al., 2017)

### Stimuli types

The test uses binary, multiple-choice, and pseudowords stimuli. For binary stimuli, a test-taker chooses whether he/she knows or does not know that stimulus (self-assessment). These items are low-demanding, easy to create, and efficient in testing large populations (Harrington & Carey, 2009; Pellicer-Sánchez & Schmitt, 2012). Many such items can be presented in a relatively short time frame, achieving high test precision without putting too much load on test-takers. However, binary stimuli have limitations (see Beeckmans et al., 2001; Mochida & Harrington, 2006). They include *overconfidence bias* (American Psychological Association, 2018b), i.e., participants decide by themselves whether they know the meaning, *self-report bias*, i.e. participants’ desire to appear more knowledgeable and meet expectations (Bauhoff, 2014), and *high false-alarm rate* (Mochida & Harrington, 2006).

For multiple-choice stimuli, a test-taker also initially chooses whether he/she knows or does not know that stimulus. If the “I know this word” option is selected, we present four options – a synonym and three distractors – and ask that the test-taker clarify the meaning of the stimulus.

Pseudowords are presented similar to binary and multiple-choice stimuli. If a test-taker indicates that he/she knows that word, we show a warning, but do not penalize the result.

The rationale for using three stimulus types is to capitalize on their complementary strengths and offset their individual limitations. Binary real-word stimuli allow for rapid testing with numerous items but suffer from high guessing probability, inflated false alarm rates, and self-report bias. Multiple-choice items significantly reduce guessing and enhance test reliability by providing more discriminative power among test-takers, yet they increase cognitive load and test duration. Pseudowords further minimize guessing probability and enhance item variability. Together, these stimulus types create a balanced and efficient assessment tool, maximizing reliability and validity while maintaining brevity in testing.

Currently, there is no consensus regarding the optimal proportion of real words (providing information on the respondent’s vocabulary) to pseudowords (providing control of the respondent’s attention and accuracy) in language tests (Beeckmans et al., 2001). Keuleers et al. (2015) used a ratio of 75% real words to 25% pseudowords, justifying this choice by the opportunity to “collect more responses to words and therefore more data.” They argued that using equal numbers of real and pseudowords would result in participants knowing fewer than 50% of the items (Keuleers et al., 2015, p. 1670). Lemhöfer et al. (2008) also adopted a similar proportion in their bilingual test employing a lexical decision task. Conversely, Mainz et al. (2017) used equal numbers of real and pseudowords in their lexical decision task, motivated by the need to compare reaction times and decision-making processes between these two item types. Pellicer-Sánchez & Schmitt (2012) considered 30% pseudowords a suitable compromise for their yes/no vocabulary test.

For the current study, we chose a proportion of 60-20-20 for binary, multiple-choice, and pseudowords stimuli respectively, since it strikes a good balance between information (coming from binary and multiple-choice stimuli) and control (coming from multiple-choice and pseudoword stimuli).

### Stimuli selection

We applied the following criteria for stimulus selection:

1. Binary and multiple-choice stimuli span from high- to low-frequent words, aiming to cover all language levels from beginners to native speakers. These words address general topics, do not have domain specification, and primarily represent modern Polish language. We tried to ensure that selected items represent unique Polish lexemes to minimize positive interference from another language by appealing to Polish dictionaries. Exceptions are made only for the most difficult, low-frequency items, e.g., *burłak* (burlak), *żyrandol* (girandole).
2. The synonyms used as multiple-choice options are hyponyms, equivalents, or close collocates of the stimuli. These synonyms have higher frequencies than the stimuli, so if test-takers know a stimulus, they most likely know its synonym as well. In some cases, synonyms are presented as two-word phrases.
  2a Distractors in multiple-choice stimuli are semantically distant from the stimuli and represent the same part of speech, reducing the guessing probability.
3. Pseudowords were randomly chosen from Imbir et al. (2015) list, being judged by participants as good examples of pseudowords.

Initially, we chose stimuli based on frequency provided in plTenTen19 web corpus (Lexical Computing CZ, n.d.; Jakubíček et al., 2013). Once data is collected, the test is going to self-calibrate based on responses, not frequencies. It is a popular index for selecting stimuli (see VST and its adaptations (Nation, 2024)). Although some studies showed that not the frequency alone but print exposure, education, source corpus, and lexical features impact vocabulary size estimation (Hashimoto, 2021; Kuperman & Van Dyke, 2013). However, Schmitt (2014) noted that with the decrease in frequency, the gap between receptive and productive vocabularies increases and Monaghan et al. (2017) argued that print exposure is the main contributor to variation in both frequency and vocabulary learning. In the present study, frequency is used for initial stimuli selection.

To extract stimuli, we applied plTenTen19 Polish web corpus in SketchEngine (Lexical Computing CZ, n.d.; Jakubíček et al., 2013). Along with a huge volume (> 18 million unique words and lemmas, > 13 M. documents), it contains a wide range of sources, including websites, titles, documents, wiki pages, etc., providing recent collocations and contextual usage. For the initial item pool, we also used the age of acquisition as a reference to the word complexity (Imbir, 2016). All selected items were further cross-checked using Polish dictionaries (PWN, https://sjp.pwn.pl/; WSJK, https://wsjp.pl/), and the national corpus (NKJP, https://nkjp.pl/).

The stimuli list was compiled in a stepwise fashion:

1. Initial test-retest compilation that includes inspection by the native language linguist
2. Pre-test on the small sample (up to 50 test-takers)
3. Piloting of the item pool at the website.

All test versions and revisions are summarized in Table 1. The pre-final (3rd) set was published on the website https://myvocab.info/pl, authors’ personal Facebook pages, and Reddit platform to collect representative dataset.

**Table 1:** The changes made in the stimuli set in the course of the pilot study

Set version	Total stimuli (incl. pseudowords)	Binary questions	Multiple-choice questions	Pseudowords
1^st^	233	146	49	38
2^nd^	290	176	76	38
3^rd^	291	181	72	38
4^rd^	233	140	55	38

### Scoring

The results of the test consist of two estimates: test-takers’ ability (latent variable), i.e., level of a trait (see Nevin et al., 2015), and attention index (a measure of trustworthiness of the result). We obtained an estimate of test-takers’ ability in logit units, which is a standard for any IRT-based test. Although these units are optimal for psychometric research, they are not easy to understand for the general public. We therefore converted it from logits to words using the logit function: y = a/[1+exp(-b*(x-c))], where *x* – test-takers’ ability in logits, *y* – vocabulary size in words, and *a, b, c* – conversion coefficients. We obtained the coefficients from fitting stimuli rank (their order from high to low frequency based on plTenTen19 corpus data) vs. stimuli difficulty (estimated from the test) using the same function.

The intuition for this conversion is the following. If we take a stimulus with a difficulty equal to a test-taker’s ability, the probability that the test-taker knows that word is 50%. If we order all words from most common to least common, that stimulus (which ended up with number *N* in the list) would split all words into two parts. The more common part (with word numbers <*N*) should contain some words that the test-taker does not know. However, the less common part (with word numbers >*N*) should also contain some words, which the test-taker does know. These two groups of words cancel each other. As a result, it is possible to say that the test-taker knows approximately *N* words. Following one of the largest Polish dictionaries – the PWN orthographic dictionary (https://shorturl.at/0Qqfb) – we assumed the total number of Polish words to be 140,000. Thus, we set coefficient *a* to 140,000, bounding the estimated vocabulary size between 0 and 140,000 words.

The attention index, which measures the trustworthiness of the results, was calculated using a formula (*x*+*y*)/(*ax*+*ay*), where *x* represents the number of pseudowords marked as unknown, *ax* is the total number of presented pseudowords, *y* is the number of multiple-choice test words defined correctly, and *ay* is the total number of presented multiple-choice questions. The attention index reaches 100% when a test-taker responds “I don’t know” to all pseudowords, and chooses correct definitions of all multiple-choice test words in the test. For individual tests, we did not use the attention index to correct (penalize) the estimation of vocabulary size. We considered all results with an attention index below 70% as not trustworthy and did not include them in the estimation of test word difficulties. We also filtered such results out when we aggregated test results.

### Validation study

#### Data analysis

The data underwent pre-processing, where missing values and outliers were removed using a standard cleaning procedure (mean ± 2 SD). We filtered out the results of test-takers who guessed too much (attention index below 70%) and whose test duration was less than 60 s. Due to the outsourced nature of our data collection, we did not have full control over participants’ self-reported demographic information, including age and language nativeness. This occasionally resulted in implausible entries, such as vocabulary scores inconsistent with known acquisition patterns or improbable ages (e.g., 0 or 100 years). To address this, we applied a data-cleaning procedure in two steps. First, we excluded participants whose reported age was below 7 years, considering ages below 7 as unreliable for consistent self-report or valid test performance. Second, to filter likely invalid nativeness data, applying a standard outlier cleaning procedure based on the mean ± 2SD rule within each group (native vs. non-native). It allowed us to identify and exclude cases where the vocabulary score was either unreasonably high for self-identified non-native speakers or unreasonably low for native speakers. After cleaning, the maximum score among non-native participants was 23,394, while the minimum score for native speakers was 19,556, which we consider plausible within the test’s difficulty range and scoring structure.

Specifically, we counted responses occurring within 5 min of each other (as more than 95% of test attempts were completed within this time), for which the reported age and native/learner status matched. We estimated that up to 10% of the data might have come from respondents who took the test multiple times. We did not exclude these responses from the analysis; however, we believe this issue does not significantly affect the results.

Variables were visually and statistically assessed for normality. Shapiro–Wilk’s test was conducted to assess the normality of the distribution of key variables. Pearson’s correlation was applied to explore relations between three key variables: vocabulary size, age, and attention index. To establish the fitness of the stimuli selected, the Rasch model was built to calculate infit outfit statistics for each stimulus and participant, and to reveal how well the test predicts the participant’s ability. Finally, an independent sample *t* test was performed to observe differences in vocabulary size between native and non-native Polish speakers.

#### Participants

The participants were recruited via the Riddle platform, Facebook, and personal invitations. After applying a rigorous data-cleaning pipeline, we retained 656 high-quality observations out of an initial 1475 participants (Table 2). Specifically, we removed participants who scored less than 75 on the attention index (meaning that they marked too many pseudowords as known and chose too many wrong definitions of multiple-choice stimuli), and those who took too little (< 30 s) time for the test. We chose to keep the results of 4% of respondents who took a significant time (> 6 min, or >12 s per stimulus) doing the test, since additional analysis showed that their results do not differ from the rest. This filtering process, though substantial, ensured the quality of our data for further statistical analysis. The native group is larger (417 vs. 239) and around 8 years older (see Table 4 below) than the non-native one.

**Table 2:** Sample size and characteristics of norm sample and two subsamples: Polish and non-Polish speakers

		Samples	
All		Polish	Non-Polish
*N*	656	417	239
M_age (SD)	30.5 (11.7)	33.4 (12.2)	25.3 (8.6)
Age range	8–66	12–66	8–49

#### Procedure

Participants took the test either online from personal computers or smartphones. The study had no time limits (Mtest_dutation ≈ 2.5 min). First, participants were instructed that they would see real and pseudowords and their task was to decide whether they knew the item. We specified “knowing” as “the ability to provide at least one of the word meanings”. Then, the participant responded to the question “Do you know the word on the screen*?*” (“Czy Państwo znają słowo na ekranie?”) and choose between two options: “I know” (“znam”) and “*I don’t know”* (“nie znam”).

Multiple-choice questions were linked to particular items from the pool and presented similarly to binary ones. If the participant responded “I don’t *know* (”nie znam”) to the binary question, the stimulus was marked as “unknown” and the test continued. But if the test-taker responded “I know” to the multiple-choice item, s/he was further obliged to “*define the word meaning*” (“określ znaczenie słowa”) by selecting one out of four items. All items were randomized.

After completing the test, participants were requested to provide their age and indicate whether their native language was Polish or non-Polish. They were also asked to confirm if they answered honestly; responses from those who did not check this box were not counted. Following this, participants received their results, which included an estimate of their receptive vocabulary size in words and statistics comparing native and non-native Polish speakers.

## Results

### Rasch analysis

To evaluate the precision of PVST, quality of stimuli, and participants’ performance, we ran a dichotomous Rasch model using the TAM package in R (Robitzsch et al., 2020) and following the guideline of Wind & Hua (2021). The Rasch equation serves to validate the psychometric test, calculating an interval-level estimation of a person’s ability, i.e., accuracy of test performance, and items’ difficulty on a linear scale (Reise & Waller, 2009; Wind & Hua, 2021). This method has already been used for vocabulary size test validations (Akase, 2022; Beglar, 2010; Runnels, 2012).

Table 3 summarizes logit-scale calibrations, SDs, and data fitness based on 1056 observations of 252 stimuli items. Average values of model fit statistics indicate a high degree of reliability with outfit and infit mean square (MSE Mean) statistics around 1.00, and standardized outfit and infit values close to 0.00. High values of the reliability of separation, which is similar to Cronbach’s alpha, demonstrate PVST high precision in the separation of test-takers based on their language proficiency and confirm that the latent trait estimates accurately reflect true individual differences (in both stimuli and respondents) with minimal measurement error. PVST demonstrated excellent precision with a STRATA value of 9.5, indicating the capacity to reliably differentiate respondents into approximately nine statistically meaningful levels.

**Table 3:** The Rasch model summary

Statistics	Items (SD)	Person (SD)
Logit scale location mean	– 1.51 (2.66)	.18 (3.12)
Outfit MSE mean	.81 (.25)	.71 (.37)
Infit MSE mean	.91 (.14)	.83 (.29)
Std. outfit mean	– 1.35 (1.99)	–.14 (1.15)
Std. infit mean	–.54 (1.05)	–.58 (.93)
Reliability of separation	.96	.95

*Note. MSE - mean squared error; Std. - standardized

**Fig. 1:**
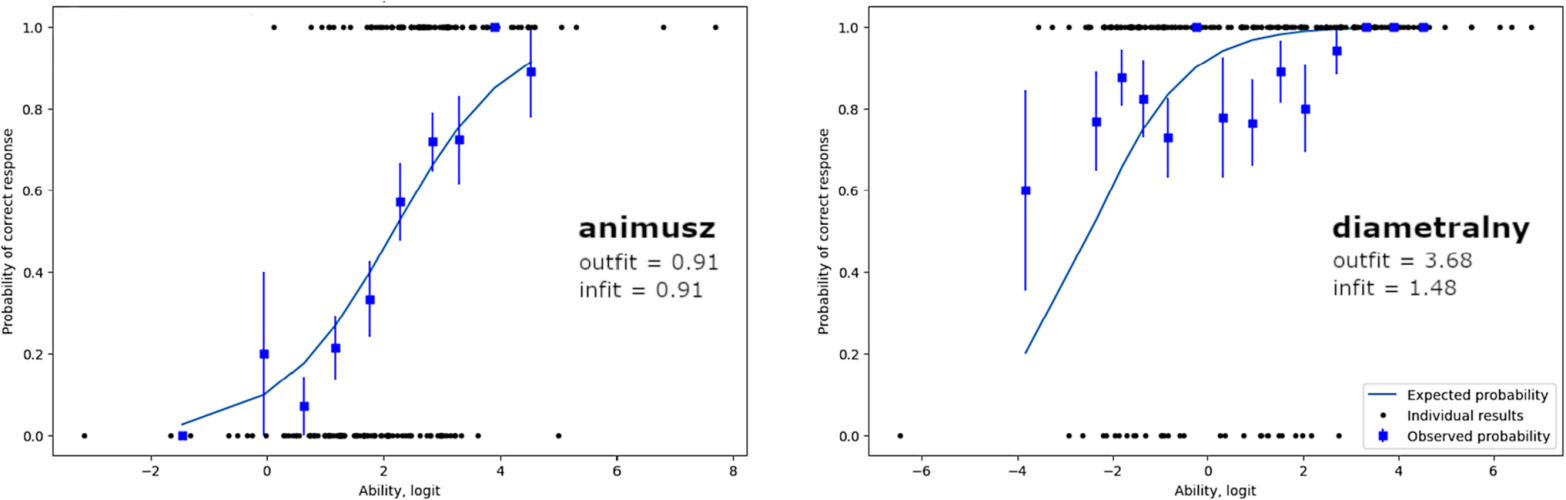
Item response curves for a high-quality (“animusz”) and low-quality (“diametralny”) test item

To assess the quality of individual test items, we plotted participants’ response curves (Fig. 1). We found that outfit and infit metrics well described the quality of the fit, i.e., how close the measured probabilities of test-takers to know a test item to the expected ones. For items with outfits and infits close to 1, the item response curves showed a very good fit (Fig. 1, left panel) while high infit and outfit reveal deviation from the measured probability (Fig. 1, right panel). After the Rasch analysis, we excluded items with infit/outfit values exceeding 1.3 and *z*-score > 2.0 (Nevin et al., 2015; Wind & Hua, 2021), leaving 195 words (with addition of 38 pseudowords).

**Fig. 2:**
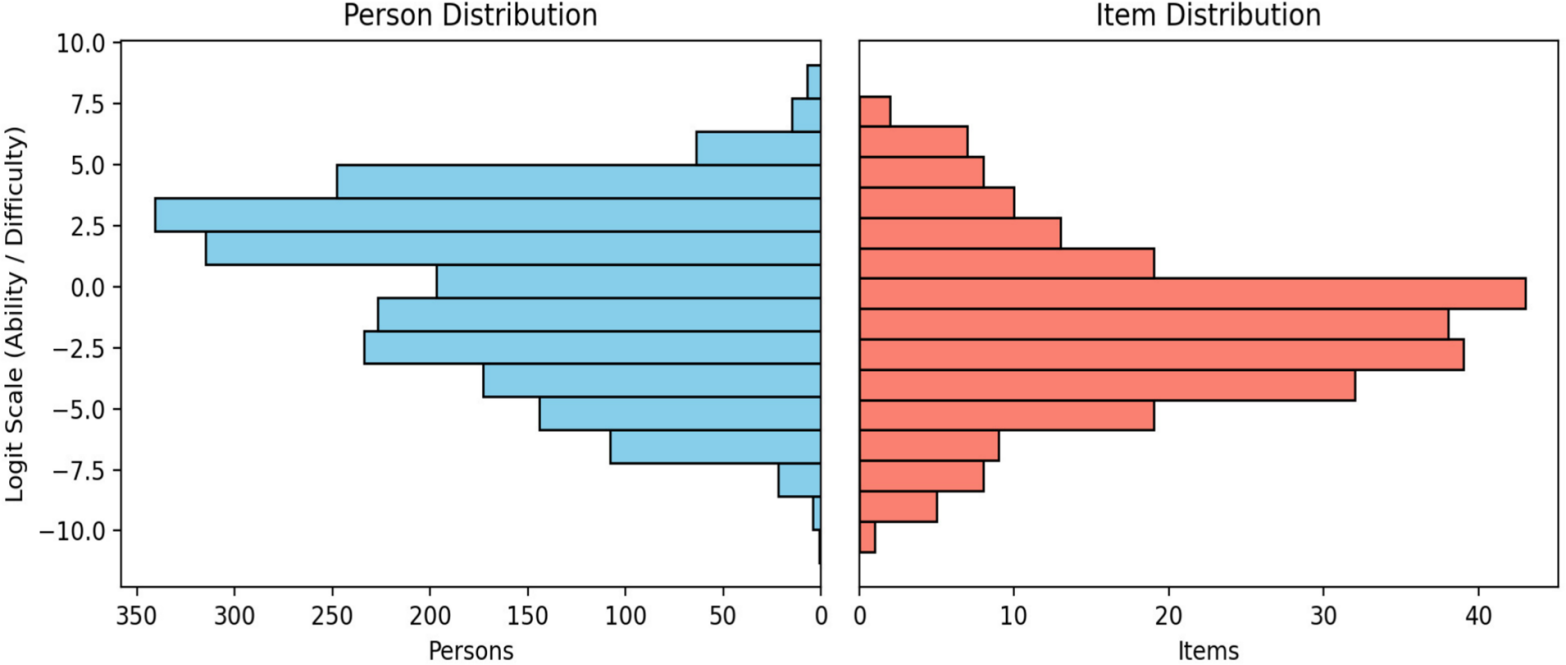
Item-person map

To visualize the quality of the stimuli bank, we can plot item-person (or Wright) map (see Fig. 2). It shows distributions of both respondents (on the left) and stimuli (on the right), plotted along the same logit scale, which becomes ability for respondents and difficulty for stimuli.

The map demonstrates that for every ability level, from beginner learners on the bottom to proficient native speakers on the top, the stimuli bank has items with corresponding levels of difficulty.

#### Vocabulary size of Polish and non-Polish speakers

Table 4 demonstrates the results of the independent samples t-test comparing two groups: native and non-native speakers.

**Table 4:** Between-group differences in age, test duration, accuracy, and vocabulary size

					95% CI Cohen’s *d*	
Variable	*df*	*t*	Effect size Cohen’s *d*	*p*	Lower	Upper
Duration	654	–.191	–.015	.849	–.175	.144
Attention index	654	–.503	–.041	.615	–.200	.118
Age	654	– 9.071	–.736	<.001	–.900	–.572
Vocabulary size	654	– 44.746	– 3.630	<.001	– 3.882	– 3.376

There were no differences between groups in test duration (*t* = –.191; *d* = –.015; M_general_ = 113.18 s; SD_general_ = 34.21) and attention index (*t* = –.503; *d* = –.041; M_general_ = 90.42; SD_general_ = 8.04), suggesting both groups are equal in their test performance. The non-native group was significantly younger than the native one (*t* = – 9.071; *d* = –.736); the vocabulary size of Polish speakers was significantly larger than those of non-Polish speakers (*t* = – 44.746; *d* – 3.630).

Table 5 reflects a ten times larger vocabulary size (*M* = 75.125; *SD* = 23.055) and a wider range (range, 19.556–122.693) of native speakers’ compared to non-speakers’ (*M* = 7.165; *SD* = 5.828; range, 646–23.394).

**Table 5:** Differences between Polish and non-Polish speakers in vocabulary size

Variable	Native language	N	M	SD	Range	*p*
Vocabulary size	Polish	417	75.125	23.055	19.556–122.693	<.001
	Non-Polish	239	7.165	5.828	646–23.394	<.001

**Fig. 3:**
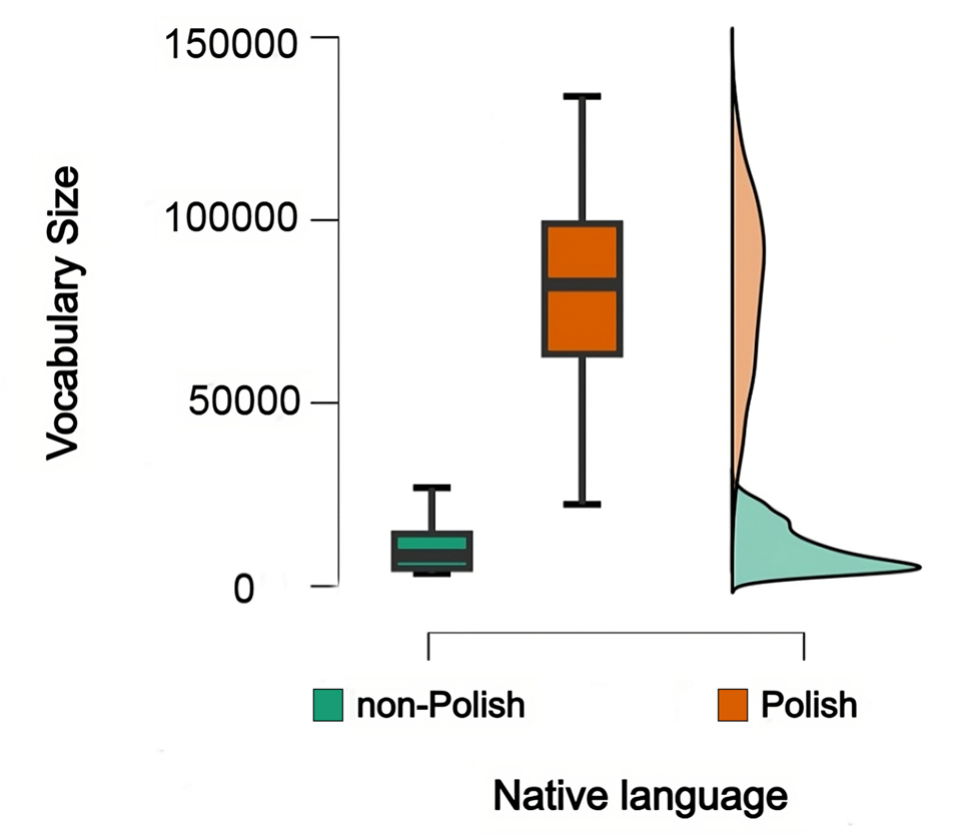
Vocabulary size distribution by native and non-native Polish speakers

The distribution of native Polish speakers’ vocabulary (Fig. 3) is close-to-normal (skewness –.167) while non-natives’ distribution is left-skewed (skewness.882) (Fig. 3, right panel) and more dense (Fig. 3, left panel).

These findings suggest that PVST is well fitted to measure vocabulary size and distinguish speakers based on language proficiency level in a relatively short time (up to 2 min) and with a low cognitive load (mean attention index ~ 90).

### Pearson’s correlation

Table 6 reveals strong positive correlations between vocabulary size and age (*r* =.496; *p* <.001), vocabulary size and attention index (*r* =.133; *p* <.001), and between test duration and age (*r* =.187; *p* <.001); moderately strong correlation between age and attention index (*r* =.085; *p* <.05), and strong negative link between duration and attention index (*r* = –.153; *p* <.001).

**Table 6:** Correlations between key variables

Variable	1	2	3	4
1. Age	—			
2. Vocabulary_size	*n* = 656 *r* =.496*** Fisher’s *z* =.544	—		
3. Attention index	*n* = 656 *r* =.085* Fisher’s *z* =.086	*n* = 656 *r* =.133*** Fisher’s *z* =.134	—	
4. Duration	*n* = 656 *r* =.187*** Fisher’s *z* =.189	*n* = 656 *r* =.068 Fisher’s *z* =.068	*n* = 656 *r* = −.153*** Fisher’s *z* = –.154	—

Note. *p <.05, **p <.01, ***p <.001

For native speakers, the correlation between the vocabulary size and age is significant (*n* = 417, *r* =.494, *p* <.001), while for non-native speakers it is weak (*n* = 239, *r* =.137; *p* <.34).

### Vocabulary size as a function of age

**Fig. 4:**
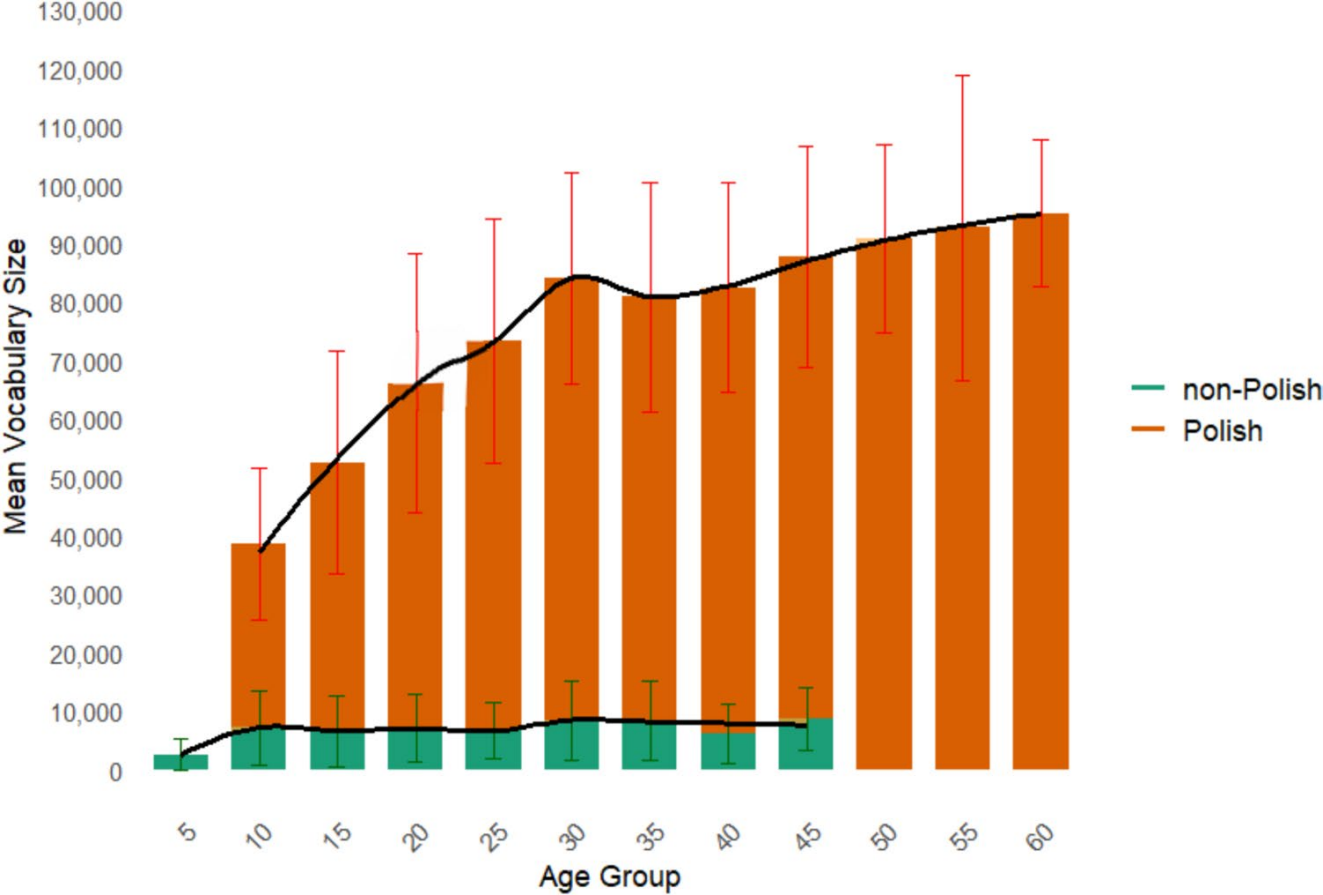
Vocabulary size changes in native and non-native speakers by age groups

Finally, to explore the impact of the native language and age on vocabulary size, we clustered participants into eleven age groups and calculated means for each group. Figure 4 demonstrates a rapid growth in vocabulary size by native speakers from 10 to 30 years old and slowing down after 30; non-Polish speakers, in turn, do not show such a tendency, having steady vocabulary size from 8 to 45 years old. The variation in vocabulary size within clusters is 2.565 to 8.566 for non-natives and 38.493 to 95.167 for native Polish speakers.

## Discussion

The main goal of the current study was to develop and pilot a novel adaptive Polish Vocabulary Size Test (PVST) using item response theory and computerized adaptive testing.

- **RQ 1.** Is the item pool used in PVST sufficiently representative for evaluating the receptive vocabulary size of Polish language users?

To ensure PVST representativeness, the item pool includes words from various parts of speech, spanning a range of frequencies and primarily rooted in Polish, with rare exceptions for low-frequency loanwords.

PVST demonstrates strong content validity (Messick, 1988) (Table 3), confirmed through Rasch modeling and strict cut-off criteria. We excluded items with infit/outfit values exceeding 1.3 and *z*-score > 2.0 (Nevin et al., 2015; Wind & Hua, 2021), reducing the initial item bank from 252 to 195 stimuli (excluding pseudowords).

The final PVST set demonstrates moderate overall difficulty (M_xsi_ = − 1.51; mean SE_xsi_ =.35). Item difficulty ranges from – 6.85 (wieczór – evening) to 5.60 (jedlca – guard, tsuba) (see Fig. 2, right panel). The item pool is skewed towards easier items, i.e. below 0, deviating from the typical range of − 3 to 3 (Embretson & Raise, 2000), revealing the imbalance of a stimulus set.

Developing a balanced item pool is inherently challenging for vocabulary size tests (Read, 2023; Stoeckel et al., 2021; Webb, 2021). Vocabulary knowledge exists on a continuum, which makes it difficult to compile a universal set of items that accurately reflects all language users’ lexicons. Nevertheless, the use of adaptive testing based on IRT allows for more detailed insights into participants’ word knowledge and mitigates some challenges in item selection (Embretson & Reise, 2000; Stoeckel et al., 2021).

Overall, the results of Rasch modeling align with prior research (Akase, 2022; Beglar, 2010; Bohn et al., 2024; Derrah & Rowe, 2015; Runnels, 2012), demonstrating PVST’s methodological robustness and high replicability. However, while the item pool represents a range of word difficulties, its skew towards easier items may limit the test’s ability to differentiate advanced vocabulary users. Future refinements should aim for a more balanced difficulty distribution to enhance representativeness across proficiency levels.

- **RQ 2.** Does the methodology used effectively capture underlying cognitive processes associated with vocabulary knowledge?

Current study employed item response theory (IRT) (Embertson & Reise, 2009; Reise & Waller, 2009) to develop a robust tool to assess receptive vocabulary size in Polish. High infit/outfit scores for both items and persons (Table 3) suggest that PVST targets vocabulary knowledge rather than unrelated cognitive constructs. This capability is a key principle of CAT, where the selection of upcoming items depends on participants’ previous responses.

PVST sensitivity to cognitive performance was further demonstrated through Pearson’s correlation. Vocabulary size was positively correlated with attention index (*r* =.133, *p* <.001) while attention index is negatively connected to the test duration (*r* = −.153, *p* <.001). These findings indicate that test-takers’ with higher attention tend to perform better and complete the test more quickly. On average, participants with higher attention scores (M_attention_index_ = 90) completed the test within 2 min. Thus, PVST effectively captures the latent construct of receptive vocabulary knowledge while minimizing cognitive burden.

As expected, PVST demonstrates precision in measuring receptive vocabulary knowledge within a short duration and with minimal cognitive load. This aligns with prior findings by Embretson & Reise (2000), who showed that shorter adaptive tests could produce data comparable in accuracy to longer assessments. In this study, the high reliability of PVST, combined with its brief administration time and strong attention index, further supports its structural validity.

- **RQ 3.** Is PVST reliable across different age groups, native and non-native speakers?

PVST demonstrates stability in distinguishing Polish and non-Polish speakers (Table 5), with native speakers exhibiting much larger vocabulary size compared to non-native ones (75.125 vs. 7.165), aligned with prior findings (Coxhead et al., 2014; Golovin, 2015). The vocabulary sizes of non-native speakers showed a tighter distribution (SD = 5828 words; range = 646–23,394 words) compared to the broader variability observed among native speakers (SD = 23,055 words; range = 19,556–122,693 words). This suggests greater individual differences in vocabulary breadth within the native speaker group. The PVST is also sensitive to age-related vocabulary size trends in native speakers, revealing vocabulary growth from ages 10 to 30, followed by a slight decline from 30 to 35 and then continuation of growth from 35 to 60.

These findings are consistent with those revealed in the studies in other languages (Brysbaert et al., 2016; Keuleers et al., 2015; Vermeiren et al., 2023). In contrast, no significant correlation between age and vocabulary size was observed among non-native speakers. This supports prior results indicating that vocabulary acquisition in this group is primarily driven by print exposure and practice but not age (San Mateo-Valdehíta & de Diego Criado 2021; Schmitt, 2014; Webb, 2005). Our findings suggest that PVST’s methodology is well suited for assessing vocabulary knowledge and reliably differentiates language proficiency among Polish language users.

In summary, the PVST effectively captures differences in vocabulary size between native and non-native speakers while demonstrating sensitivity to age-related trends in vocabulary acquisition. It aligns with established vocabulary test standards and replicates widely recognized findings. Grounded in item response theory and computerized adaptive testing, PVST is efficient, cognitively nondemanding, precise, and easy to administer.

The test’s accessibility through an online interface further enhances its usability. Incorporating gamification elements, such as interactive features, encourages user engagement and supports test completion. These features make PVST a versatile tool with potential applications in various fields, including educational research, language instruction, psycholinguistics, and psychology.

## Limitations

The pilot study faced several limitations. First, remote data collection led to self-reported age and language information, resulting in approximately 50% data loss after cleaning. The study’s unsupervised nature also raises the possibility of test retakes and cheating, though the remaining data were sufficient for robust analysis. Additionally**,** the stimulus list, while balanced based on Rasch analysis, may still require refinement to improve item selection. The observed vocabulary size of Polish speakers (*M* = 75.125) significantly differs from findings of Brysbaert et al. (2016), Guasch et al. (2023), and Keuleers et al. (2015) but align with Golovin (2015). It is likely due to statistical and lexical boundary differences: an upper bound of 140,000 words is based on the PWN dictionary.

## Declarations

### Preregistration

The research was not preregistered before the data collection.
