# Correction: The Polish Vocabulary Size Test: A novel adaptive test for receptive vocabulary assessment

**DOI:** 10.3758/s13428-025-02825-w

**Published:** 2025-09-17

**Authors:** Danil Fokin, Monika Płużyczka, Grigory Golovin

**Affiliations:** 1 University of Warsaw, Warsaw, Poland; 2 Independent, San Jose, CA USA

**Correction: Behavior Research Methods (2025) 57:254**

10.3758/s13428-025-02775-3

The original online version of this article was revised: The author with the surname Płużyczka—was misspelled as Plużyczka. The misspelling has been corrected.

